# Altered synaptic plasticity at hippocampal CA1–CA3 synapses in Alzheimer's disease: integration of amyloid precursor protein intracellular domain and amyloid beta effects into computational models

**DOI:** 10.3389/fncom.2023.1305169

**Published:** 2023-12-07

**Authors:** Justinas J. Dainauskas, Paola Vitale, Sebastien Moreno, Hélène Marie, Michele Migliore, Ausra Saudargiene

**Affiliations:** ^1^Neuroscience Institute, Lithuanian University of Health Sciences, Kaunas, Lithuania; ^2^Department of Informatics, Vytautas Magnus University, Kaunas, Lithuania; ^3^Institute of Biophysics, National Research Council, Palermo, Italy; ^4^Université Côte d'Azur, Centre National de la Recherche Scientifique (CNRS), Institut de Pharmacologie Moléculaire et Cellulaire (IPMC), Valbonne, France

**Keywords:** Alzheimer's disease, amyloid precursor protein intracellular domain, amyloid beta, synaptic plasticity, NMDA receptor, GluN2B-NMDA receptor subunit, Schaffer collateral synapses, CA1 pyramidal neuron

## Abstract

Alzheimer's disease (AD) is a progressive memory loss and cognitive dysfunction brain disorder brought on by the dysfunctional amyloid precursor protein (APP) processing and clearance of APP peptides. Increased APP levels lead to the production of AD-related peptides including the amyloid APP intracellular domain (AICD) and amyloid beta (A*β*), and consequently modify the intrinsic excitability of the hippocampal CA1 pyramidal neurons, synaptic protein activity, and impair synaptic plasticity at hippocampal CA1–CA3 synapses. The goal of the present study is to build computational models that incorporate the effect of AD-related peptides on CA1 pyramidal neuron and hippocampal synaptic plasticity under the AD conditions and investigate the potential pharmacological treatments that could normalize hippocampal synaptic plasticity and learning in AD. We employ a phenomenological N-methyl-D-aspartate (NMDA) receptor-based voltage-dependent synaptic plasticity model that includes the separate receptor contributions on long-term potentiation (LTP) and long-term depression (LTD) and embed it into the a detailed compartmental model of CA1 pyramidal neuron. Modeling results show that partial blockade of Glu2NB-NMDAR-gated channel restores intrinsic excitability of a CA1 pyramidal neuron and rescues LTP in AICD and A*β* conditions. The model provides insight into the complex interactions in AD pathophysiology and suggests the conditions under which the synchronous activation of a cluster of synaptic inputs targeting the dendritic tree of CA1 pyramidal neuron leads to restored synaptic plasticity.

## 1 Introduction

Alzheimer's disease (AD) has a long preclinical stage and, before any clinical symptoms appear, pathological processes are observed in the hippocampus and entorhinal cortex, key brain structures responsible for memory encoding and retrieval. AD cannot be prevented, halted, or cured today, and new interdisciplinary ways are urgently needed for the understanding and treatment of this devastating disease. Recent experimental evidence supports the fundamental role of AD-related peptides early in the pathology: in particular, the most widely studied amyloid beta (A*β*) and the less investigated amyloid precursor protein (APP) C-terminal peptide (AICD). Their differential effects on synaptic function and intrinsic excitability of hippocampal CA1 pyramidal neuron at a single cell level are currently being investigated. However, the impact and complex interaction effects of A*β* and AICD on hippocampal synaptic plasticity and CA1 neuron activity remain largely unknown.

It is widely believed that, before any ongoing tau or neuroinflammation pathologies, the first molecular events occurring in the AD brain are alterations of APP processing and/or clearance of APP peptides. This leads to the well-documented alteration in levels of A*β*, which readily aggregates (Haass and Selkoe, 2007). There is also evidence that the levels of AICD, the production of which is intimately linked with A*β*, processing, are also elevated in early AD (Ghosal et al., 2009; Rajão-Saraiva et al., 2023). Accumulating experimental evidence suggests that each peptide plays a role in modifying hippocampus function in the early stages of the disease. AICD has a strong impact on synapse function, as recently shown by Pousinha et al. (2017) and on intrinsic excitability (Pousinha et al., 2019). Increased levels of AICD, as previously observed in AD mouse models and human patients, enhance GluN2B-NMDAR contribution, overactivate SK channels, and strongly perturb long-term potentiation (LTP), but spare long-term depression (LTD) in CA1 pyramidal neurons. Partial antagonism of GluN2B-NMDAR rescues LTP in early AD.

Oligomeric forms of A*β* prevented induction of LTP in hippocampal cultured neurons (Opazo et al., 2018). Incubation with oligomeric A*β* led to a dose-dependent activation of calcium/calmodulin-dependent kinase II (CaMKII) via GluN2B-NMDAR (Opazo et al., 2018). CaMKII is a key protein for LTP expression, activated directly by *Ca*^2+^ influx through GluN2B-NMDAR and leading to phosphorylation of synaptic proteins and increase in the number of active AMPARs or their single-channel conductance (Shipton and Paulsen, 2014; Park et al., 2021; Yasuda et al., 2022). Oligomeric A*β* increased CaMKII overall activity, prevented its subsequent *T*286 autophosphorylation by plasticity-inducing stimulation, and led to the LTP-mediated immobilization of CaMKII at dendritic spines and diffusional trapping of AMPARs. In addition, oligomeric A*β* caused dendritic spine loss in a GluN2B-NMDAR-dependent manner (Opazo et al., 2018). CaMKII inhibitors KN93, tatCN21, and specific GluN2B-NMDAR antagonist ifenprodil completely rescued A*β*-induced inhibition of LTP by preventing CaMKII overactivation and dendritic spine loss (Opazo et al., 2018). The results demonstrated that A*β* prevents LTP induction by activating CaMKII in a GluN2B-NMDAR-dependent manner.

This study aims at explaining early hippocampus-dependent learning and memory deficits induced by increased levels of AICD and A*β*, a condition that mimics early AD. To model the effects of AICD and A*β* on excitatory neuron activity, we focused on recent publications analyzing the acute effect (1–5 h) of peptide-delivered *ex-vivo* at physiopathologically relevant concentrations (nanomolar range; Abramov et al., 2009; Pousinha et al., 2017, 2019; Opazo et al., 2018; Taylor et al., 2021).

We developed a data-driven *in silico* model of the hippocampal CA1 pyramidal neuron under AD conditions, with the main objectives being to incorporate the effects of AICD and A*β* into computational models of CA1 pyramidal neuron and hippocampal synaptic plasticity. The goal of this study was to explain complex interactions of synaptic and cellular-level mechanisms of altered hippocampal function that leads to impaired learning and progressive irreversible memory loss in AD, and finally, to identify and assess potential targets for innovative pharmacological treatment of AD.

## 2 Methods

We used a modified NMDAR subunit-dependent voltage-based model of synaptic weight change at hippocampal CA3–CA1 synapses (Dainauskas et al., 2023) to investigate the effect of elevated levels of A*β* and AICD. We embedded this model into a multicompartmental CA1 pyramidal neuron model (Peng et al., 2016; Migliore et al., 2018) to study the influence of AICD and of A*β* on synaptic weights within a cluster of randomly distributed CA3–CA1 synapses onto apical dendrites of a CA1 neuron in the stratum radiatum (SR) region. We modeled LTP and LTD, induced by high and low frequency stimulation, respectively, and the alterations due to the increased levels of AD peptides. Finally, we analyzed the effect of GluN2B-NMDAR function on the synaptic properties and a possible pharmacological treatment to restore normal synaptic function in AD.

### 2.1 Synapse model under AICD and A*β* conditions

We employed a phenomenological voltage-dependent NMDAR-based synaptic plasticity model, developed in our previous study (Dainauskas et al., 2023) (ModelDB accession number 267680), and extended it to incorporate the effects of AICD and A*β* in AD. The model relies on the functioning of the GluN2A-NMDAR and GluN2B-NMDAR subunits as the separate mediators of LTD and LTP, respectively. It is assumed that LTP is mainly dependent on GluN2B-NMDAR (Morishita et al., 2007; Andrade-Talavera et al., 2016; Pousinha et al., 2017), and GluN2A-NMDAR is the main mediator of LTD (Morishita et al., 2007).

**Figure 1:**
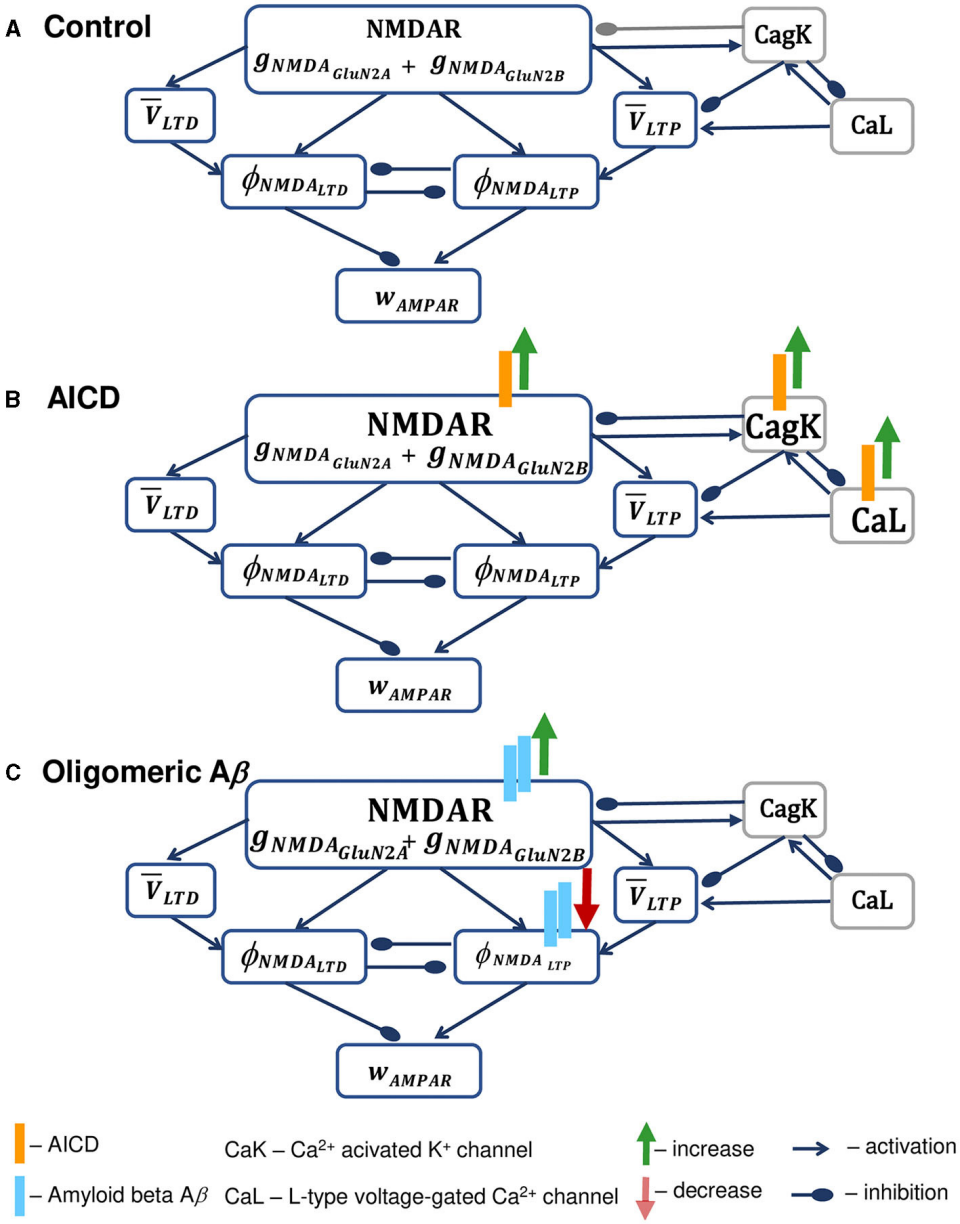
Schematic diagram of the synapse model in control conditions **(A)**, under the increased AICD **(B)**, and A*β* **(C)** concentrations in AD. **(A)** Presynaptic action potential activates NMDAR, composed of GluN2B-NMDAR and GluN2A-NMDAR subunits *g*_*NMD*_*A*__*GluN*2*B*__ and *g*_*NMD*_*A*__*GluN*2*A*__, and triggers LTP and LTD functions *ϕ*_*NMD*_*A*__*LTP*__ and *ϕ*_*NMD*_*A*__*LTD*__, respectively. These LTP and LTD functions *ϕ*_*NMD*_*A*__*LTP*__ and *ϕ*_*NMD*_*A*__*LTD*__ mutually inhibit each other. NMDAR contributes to the postsynaptic local membrane potential that is low-pass filtered, and the resulting LTP and LTD variables ${\bar{V}}_{LTP}$ and ${\bar{V}}_{LTD}$ are multiplied by the corresponding NMDAR-dependent functions *ϕ*_*NMD*_*A*__*LTP*__ and *ϕ*_*NMD*_*A*__*LTD*__ to form the LTP and LTD components of the AMPAR weight *w*_*AMPAR*_. NMDAR is inhibited by the activity of the nearby *Ca*^2+^-dependent *K*^+^ channels *CagK*, that are in turn triggered by the NMDAR activation and lead to the hyperpolarization of the membrane potential. In a similar manner, *Ca*^2+^-dependent *K*^+^ channels *CagK* are activated by *L*-type *Ca*^2+^ channels *CaL*. Dendritic ion channels *CagK* and *CaL* exhibit the effect on synaptic weight *w*_*AMPAR*_ indirectly through the influence on NMDAR and membrane potential filtered values ${\bar{V}}_{LTP}$ and ${\bar{V}}_{LTD}$, and are therefore presented in gray boxes. **(B)** Elevated AICD concentrations (orange bars) lead to GluN2B-NMDAR-gated channel conductance upregulation (green arrow) and increase in *Ca*^2+^-activated *K*^+^ channel *CagK* conductance (green arrow) and *L*-type *Ca*^2+^ channel *CaL* conductance (green arrow; Pousinha et al., 2017, 2019). The resulting high intracellular *Ca*^2+^ levels overactivate *Ca*^2+^-dependent *K*^+^ channels and cause hyperpolarization of the neuron membrane, thus leading to the failure in LTP induction. Increase in specific membrane conductance and in axonal, somatic, and dendritic *M*-type *K*^+^ current and decrease in axonal, somatic, and basal *Na*^+^ current are omitted. **(C)** Oligomeric A*β* (light blue bars) increases NMDAR activation (green arrow) through glutamate transmission facilitation (Abramov et al., 2009; Taylor et al., 2021) and prevents the activation of LTP protein CaMKII expressed as a phenomenological function *ϕ*_*NMD*_*A*__*LTP*__, thus leading to deficits in LTP (Opazo et al., 2018). Spine loss caused by A*β* is not indicated.

Schematic diagram of the synapse model in control conditions and under the influence of increased AICD and A*β* is presented in Figure 1. In the model, active NDMARs, consisting of GluN2B-NMDAR and GluN2A-NMDAR subunits, trigger LTP and LTD functions *ϕ*_*NMD*_*A*__*LTP*__ and *ϕ*_*NMD*_*A*__*LTD*__, respectively, that represent second messenger pathways responsible for LTP and LTD induction, such as CaMKII and phosphatase activation, in a phenomenological manner. These functions take a form of a Hill equation and are activated mainly by the postsynaptic GluN2B-NMDAR for LTP term and by postsynaptic GluN2A-NMDAR for the LTD term. Subsequently, the functions *ϕ*_*NMD*_*A*__*LTP*__ and *ϕ*_*NMD*_*A*__*LTD*__ are multiplied by the low-filtered membrane potential at a synapse location ${\bar{V}}_{LTP}$ and ${\bar{V}}_{LTD}$, correspondingly, to form the LTP and LTD components that linearly add to calculate the instantaneous change of the AMPAR weight *w*_*AMPA*_ (Figure 1A). In addition, the model diagram shows dendritic *Ca*^2+^-dependent *K*^+^ channels *CagK* and *L*-type *Ca*^2+^ channels *CaL*, that shape synaptic plasticity properties in AD. Specifically, intracellular NMDAR-mediated *Ca*^2+^ activates the nearby *Ca*^2+^-dependent *K*^+^ channels *CagK*, that may lead to hyperpolarization of the membrane potential and in turn inhibit NMDAR. Moreover, these *Ca*^2+^-dependent *K*^+^ channels *CagK* are activated by *Ca*^2+^ through *L*-type *Ca*^2+^ channels *CaL*. As dendritic ion channels *CagK* and *CaL* influence synaptic weight *w*_*AMPAR*_ indirectly through NMDAR and membrane potential filtered values ${\bar{V}}_{LTP}$ and ${\bar{V}}_{LTD}$, these elements are presented in gray boxes. The model equations are described in Supplementary material.

To study the effect of AICD on synaptic plasticity, we incorporated the AICD influence on NMDAR and ion channels following Pousinha et al. (2017, 2019). Specifically, as shown in Figure 1B, elevated AICD levels (orange bars) result in the increased GluN2B-NMDAR—gated channel conductance (green arrow), and increased *Ca*^2+^—dependent *K*^+^ channel *CagK* conductance (green arrow), increased *L*-type *Ca*^2+^ channel *CaL* conductance (green arrow). In addition, AICD leads to the increase in specific membrane conductance and axonal, somatic, and dendritic *M*-type *K*^+^ current, and decrease in axonal, somatic, and basal *Na*^+^ current. These modifications are omitted in the model diagram because the changes in channels are not observed in the proximity of a synapse. As a result of the elevated AICD levels, intracellular *Ca*^2+^ concentration rises, overactivates *Ca*^2+^—dependent *K*^+^ channels, and leads to the hypoexcitability of the neuron preventing LTP induction.

We modeled the influence of AICD by increasing conductances of GluN2B-NMDAR-gated channels by 400% (Pousinha et al., 2019); dendritic and somatic *Ca*^2+^-dependent *K*^+^ channels by 120%; somatic and dendritic *L*-type *Ca*^2+^ channels by 200%; axonal, somatic, and dendritic *M*-type channels by 120, 200, and 200%, respectively, decreasing the conductance of axonal, somatic, and basal *Na*^+^ channels by 22.2, 66.6, and 33.3%, respectively, and increasing specific membrane conductance by 180%.

Furthermore, we analyzed the influence of increasing oligomeric A*β* levels (Figure 1C). Oligomeric forms of A*β* (light blue bars) lead to the increase in glutamate transmission and higher NMDAR (green arrow) and AMPAR activations, prevention of CaMKII phosphorylation in a GluN2B-NMDAR-dependent manner (red arrow), and spine loss (Opazo et al., 2018). A critical role of GluN2B-NMDAR in oligomeric A*β*-mediated LTP impairment in hippocampal slices was also shown by Rönicke et al. (2011). A*β* oligomers also increase glutamate release probability (Abramov et al., 2009) and enhance LTD (Taylor et al., 2021).

We model the effect of A*β* by increasing the glutamate concentration by 120% (Abramov et al., 2009), that causes enhancement of LTD (Taylor et al., 2021). We reduce synaptic density to 80% (Opazo et al., 2018) and transform the GluN2B-NMDAR-dependent function for LTP component *ϕ*_*NMD*_*A*__*LTP*__ into the bell-shape function so that it remains close to zero even when GluN2B-NMDAR is activated, thus failing to trigger LTP (Equation S4, Supplementary material).

Modifications of the synaptic plasticity model parameters under AICD and A*β* conditions are given in Table 1.

**Table 1:** Modifications of parameters of synaptic plasticity model and multicompartmental CA1 pyramidal neuron model in AICD and A*β* conditions.

**Parameter**	**Change**	**Value and unit**	**References**
**AICD conditions**			
Scaling factor *K*_*GluN*2*B*_ of conductance of GluN2B-NMDAR *g*_*NMD*_*A*__*GluN*2*B*__, Equation (S14) in Supplementary material	Increased by 400%	4 (1)	Pousinha et al., 2019
Conductance of somatic *Ca*^2+^-dependent *K* channel, *CagK*	Increased by 120%	700 (600) μ*S*/*cm*^2^	Optimized following Pousinha et al. (2019)
Conductance of dendritic *Ca*^2+^-dependent *K* channel, *CagK*	Increased by 120%	700 (600) μ*S*/*cm*^2^	Optimized following Pousinha et al. (2019)
Conductance of somatic *L*-type *Ca*^2+^ channel, *CaL*	Increased by 183%	0.77 (0.42) μ*S*/*cm*^2^	Optimized following Pousinha et al. (2019)
Conductance of dendritic *L*-type *Ca*^2+^ channel, *CaL*	Increased by 200%	0.77 (0.42) μ*S*/*cm*^2^	Optimized following Pousinha et al. (2019)
Conductance of axonal *M*-type *K*^+^ channel	Increased by 120%	34 (27) μ*S*/*cm*^2^	Optimized following Pousinha et al. (2019)
Conductance of somatic *M*-type *K*^+^ channel	Increased by 200 %	82 (42) μ*S*/*cm*^2^	Optimized following Pousinha et al. (2019)
Conductance of axonal *Na*^+^ channel	Decreased to 22.2%	60 × 10^3^ (270 × 10^3^) μ*S*/*cm*^2^	Optimized following Pousinha et al. (2019)
Conductance of somatic *Na*^+^ channel	Decreased to 66.6%	69 × 10^3^ (98 × 10^3^) μ*S*/*cm*^2^	Optimized following Pousinha et al. (2019)
Conductance of basal *Na*^+^ channel	Decreased to 33.3%	60 × 10^3^ (170 × 10^3^) μ*S*/*cm*^2^	Optimized following Pousinha et al. (2019)
Specific membrane conductance of CA1 pyramidal neuron	Increased by 180%	49 (27) μ*S*/*cm*^2^	Optimized following Pousinha et al. (2019)
**A***β* **conditions**			
Glutamate concentration during synapse activation, *Glu* in Equations (S18), (S19), (S27), (S28) in Supplementary material	Increased by 120%	1.2 (1) *mM*	Abramov et al., 2009
Value of the filtered ${\bar{g}}_{NMDA_{LTP}}$ producing half activation of *ϕ*_*NMD*_*A*__*LTP*__ for LTP component inhibition in A*β* conditions, *K*_*a*_2__*LTP*__ in Equation (S4) in Supplementary material	Decreased to model prevention of CaMKII activation by A*β*	6.5 × 10^−2^ (1) μ*S*	Adjusted, following Opazo et al. (2018)
Synapse density	Decreased to 80%	0.64 (0.8) synapse/μ*m* dendrite	Opazo et al., 2018

Parameters in control conditions are indicated in parentheses.

### 2.2 Multicompartmental model of CA1 pyramidal neuron

We used a morphology reconstruction of a CA1 pyramidal neuron downloaded from: http://www.neuromorpho.org (Peng et al., 2016) (cell *fx*_*CA*1_7.*CNG*.*swc*). The channel kinetics were based on those used in previously published articles on CA1 hippocampal neurons and validated against a number of experimental findings. The model was implemented with channel kinetics used by Migliore et al. (2018) (ModelDB accession number 244688). In particular, we used a delayed-rectifier type current (*K*_*DR*_), two *A*-type potassium (*K*_*A*_) currents (for proximal and distal dendrites), a delayed type current (*K*_*D*_), three types of voltage-dependent *Ca*^2+^ currents (*CaL*, *CaT*, and *CaN*), the slow afterhyperpolarization (*AHP*) *Ca*^2+^-dependent *K*^+^ current (*kCa*), a *Na*^+^ current, and a calcium pump. The kinetics for the non-specific hyperpolarization-activated current (*Ih*), a *M*-type potassium, *K*_*M*_, and the medium afterhyperpolarization (*AHP*) *Ca*^2+^-dependent *K*^+^ currents (*CagK*) were optimized for the specific classical accommodating traces by using both the standard Run Fitter tool available in NEURON and the BluePyOpt. In particular, the genetic algorithm used calculates the best seed among 128 offspring produced during a maximum of 100 generations. We performed single-cell optimizations by running four seeds at the same time. Electrophysiological features were extracted from experimental recordings using a tool of the EBRAINS Cellular Level Modeling workflows (https://ebrains.eu/service/cls-interactive/), based on the open-source Electrophysiological Feature Extraction Library (eFEL). The optimizations were carried out simultaneously on any given set of experimental traces until they converged into a good solution (error lower than 0.3 mV using Neuron Run Fitter and three standard deviation in the case of BluePyOpt). The ionic channels were distributed on the membrane according to experimental findings. The *K*_*A*_ and *I*_*h*_ increased with distance from the soma (Hoffman and Johnston, 1999), while *K*_*D*_ decreased with the distance from the soma. Passive properties and peak conductance for each channel were adapted from their original values to qualitatively reproduce the experimental recordings used as a reference. The specific membrane capacitance (*Cm*) and the time constant of the calcium pump were included as fitting parameters during the optimization. The values for the peak conductance of each channel were independently optimized in each type of compartment (soma, axon, basal, and apical dendrites).

Starting from results reported by Pousinha et al. (2019) and using the experimental recordings from control mouse and AICD pyramidal neurons herein analyzed, we decided to implement a single cell model optimization, using one typical set of data for each phenotype. In the study by Pousinha et al. (2019), it was shown that AICD causes a decrease in the firing frequency through the enhancement of the afterhyperpolarization. In accordance with several studies, this peculiar behavior is related to the alteration of *L*-type *Ca*^2+^ channels, the *Ca*^2+^-activated *K*^+^ channels (*SK* or *CagK*) and the *Kv*7/*M* channels (Kumar and Foster, 2002). For this reason, we first implemented the model for the control condition. Then, keeping constant all the peak channel conductances not directly involved in the firing frequency alteration discussed by Pousinha et al. (2019), we optimized the model to reproduce AICD conditions by re-optimizing only passive properties and the peak conductances for *CaL*, *CagK*, *K*_*M*_, and *Na*^+^ channels. The results suggested that AICD neurons will have a lower *Na*^+^ conductance and ionic channels modulating adaptation would be at higher density, with respect to control. While searching the literature, we could only find one publication (Tamagnini et al., 2015) reporting a minimal impact of oligomeric A*β* on CA1 pyramidal excitability profile in conditions identical to the description of A*β*'s effects on synaptic function used by Opazo et al. (2018) (500 nM oligomeric A*β* application for a few hours on hippocampal slices). Under the A*β* conditions, the excitability profile of CA1 pyramidal neuron was thus not modified. Modifications of the multicompartmental CA1 pyramidal neuron model parameters under AICD conditions are given in Table 1.

We formed a cluster of 50 AMPARs and GluN2A/GluN2B-NMDARs synapses, randomly distributed on the apical dendrites of CA1 pyramidal neurons in the SR region. Synapses were defined with a distance of 100–300 μ*m* from the soma and a synaptic density of 0.8 synapses/μ*m* of dendrite (Gasparini et al., 2004; Bezaire et al., 2016).

Synapses were stimulated at 100 Hz for 1 s (LTP protocol) or at 1 Hz for 500 s (LTD protocol) as in the study by Pousinha et al. (2017). To estimate the change in the somatic postsynaptic excitatory potential (EPSP), a presynaptic stimulus was delivered before and after the conditioning stimulation, and the resulting ratio between the maximal values of the resulting EPSPs was calculated. The enhancement and partial blockade of the GluN2B-NMDAR-gated channel in AICD and A*β* conditions was simulated by varying the conductance *g*_*NMD*_*A*__*GluN*2*B*__ (Equation S14 in Supplementary material).

All simulations were carried out the NEURON simulation environment (Hines and Carnevale, 1997) integrated with Python (Van Geit et al., 2016). Optimization of the neuron parameters was performed as a parallel code executed on different high-performance computing systems: JURECA (Juelich Supercomputing Center, Germany), Galileo100 (CINECA, Italy), and Piz Daint (Swiss National Supercomputing Center CSCS).

All model files in NEURON and Python are available for public download under the ModelDB section of the Senselab database, accession numbers 2014822 and 2015000 (https://senselab.med.yale.edu/ModelDB/).

## 3 Results

We used the extended synaptic plasticity model, embedded into the multicompartmental model of a CA pyramidal neuron, to study how high levels of AICD and A*β* influence synaptic changes at CA3–CA1 synapses in AD. First, we validated the model against the experimental data on impaired synaptic plasticity for increased levels of AICD (Pousinha et al., 2017, 2019) and A*β* (Opazo et al., 2018), and then analyzed the effect of the GluN2B-NMDAR blockade to rescue LTP in conditions when AICD, A*β*, or both toxic peptides are present. In summary, we modeled the following scenarios for LTP and LTD induction at a cluster of AMPARs and GluN2A/GluN2B-NMDARs synapses on the proximal apical dendrites of CA1 pyramidal neuron:

- Control conditions.
- AD conditions:

– increased levels of AICD concentration;
– increased levels of A*β* concentration;
– increased levels of AICD and A*β*.

- AD conditions and restored synaptic plasticity:

– increased levels of AICD concentration with a partial blockade of Glu2NB-NMDAR;
– increased levels of A*β* concentration with a partial blockade of Glu2NB-NMDAR;
– increased levels of AICD and A*β* with a partial blockade of Glu2NB-NMDAR.

### 3.1 Model validation against experimental findings of impaired synaptic plasticity under AICD and A*β* conditions

**Figure 2:**
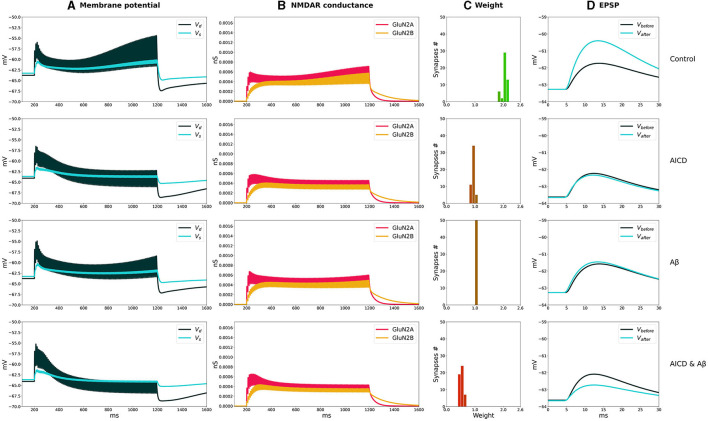
LTP induction in control, AICD, A*β*, and AICD & A*β* conditions. Presynaptic inputs were stimulated at 100 Hz for 1 s. GluN2B-NMDAR synaptic conductance was increased by a factor of 4 in AICD and AICD & A*β* conditions. **(A)** Membrane potential in soma *V*_*s*_ (cyan line) and at a single synapse *V*_*d*_ (dark green line). **(B)** GluN2A-NMDAR synaptic conductance (red line) and GluN2B-NMDAR synaptic conductance (yellow line). **(C)** Synaptic weight distribution after the LTP induction protocol. **(D)** Somatic EPSP before (black line) and after (cyan line) LTP induction protocol. Rows top to bottom: (Control) Somatic EPSP change is 183%. (AICD) Somatic EPSP change is 94%. (A*β*) Somatic EPSP change is 105%. (AICD & A*β*) Somatic EPSP change is 60%.

**Figure 3:**
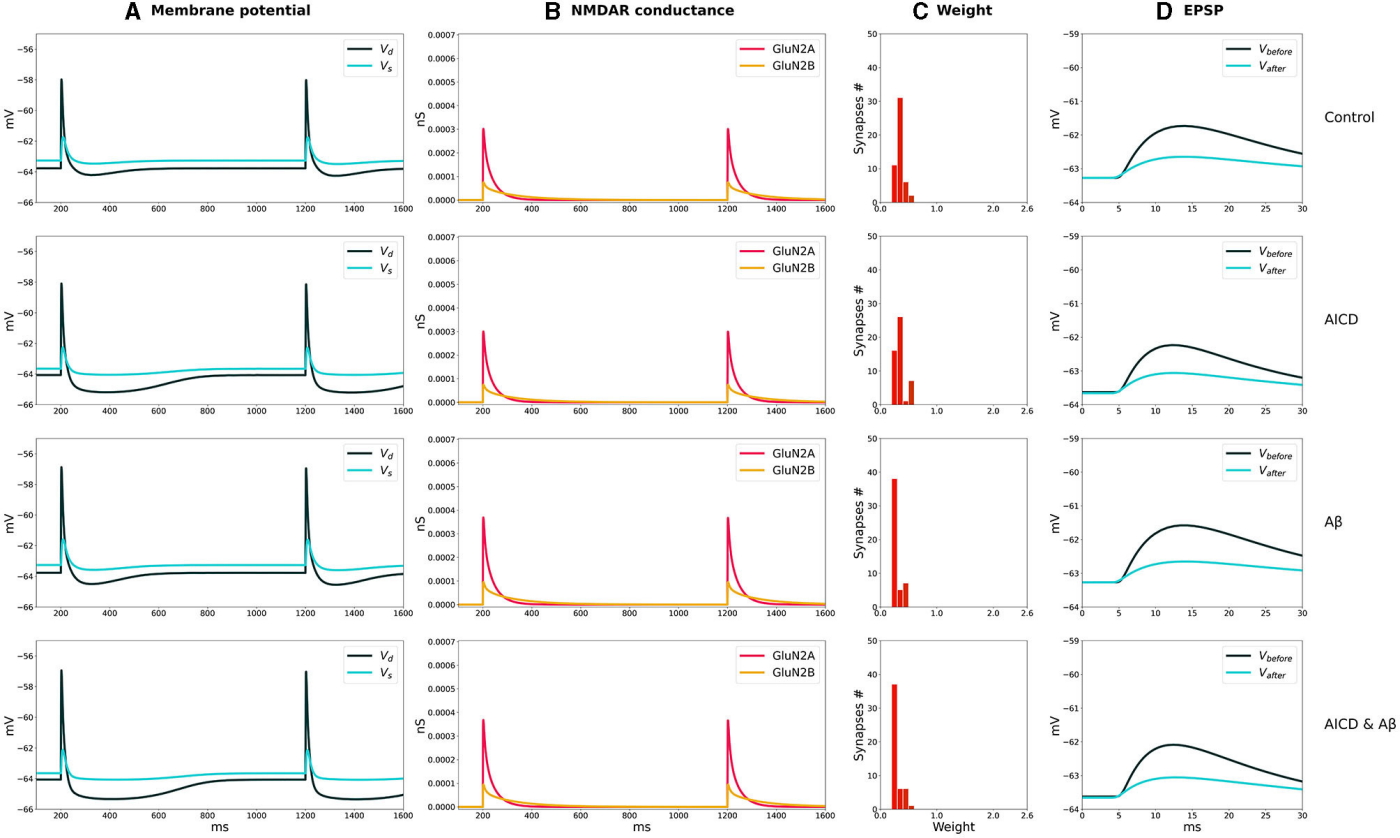
LTD induction in control, AICD, A*β*, and AICD & A*β* conditions. Presynaptic inputs were stimulated at 1 Hz for 500 s. GluN2B-NMDAR synaptic conductance was increased by a factor of 4 in AICD and AICD & A*β* conditions. **(A)** Membrane potential in soma *V*_*s*_ (cyan line) and at a single synapse *V*_*d*_ (dark green line). **(B)** GluN2A-NMDAR synaptic conductance (red line) and GluN2B-NMDAR synaptic conductance (yellow line). **(C)** Synaptic weight distribution after the LTD induction protocol. **(D)** Somatic EPSP before (black line) and after (cyan line) LTD induction protocol. Rows top to bottom: (*Control*) Somatic EPSP change is 40%. (*AICD*) Somatic EPSP change is 42%. (A*β*) Somatic EPSP change is 36%. (AICD & A*β*) Somatic EPSP change is 39%.

The distributions of synaptic weights, the traces of somatic and dendritic membrane potential, and synaptic conductances of GluN2A-NMDAR and GluN2B-NMDAR, crucial for synaptic modification induction at CA3–CA1 synapses, are presented for LTP and LTD stimulation protocols in control, AICD, A*β*, and AICD & A*β* conditions in Figures 2, 3.

In control conditions, the dendritic depolarization caused by a conditioning stimulation at 100 Hz for 1 s, activated Glu2NA-NMDAR and Glu2NB-NMDAR. During this period, the weight of each synapse in a cluster independently evolved according to the local membrane potential and NMDAR-gated synaptic conductance function. Increased Glu2NB-NMDAR triggered LTP induction and inhibited LTD induction. Synaptic weights increased up to the peak value, as shown in the histogram, and the somatic EPSP response increased by 183% (Figure 2, Control).

AICD-mediated alterations in Glu2NB-NMDAR, i.e., upregulation of *Ca*^2+^-dependent *K*^+^ channels *CagK* and *L*-type *Ca*^2+^ channels *CaL* resulted in a neuron hypoexcitability (Pousinha et al., 2019), effectively preventing synaptic plasticity (Figure 2, AICD). Increased concentration of oligomeric A*β* also impaired LTP, limiting its induction to 105% (Figure 2, A*β*). The model suggested that the observed changes were caused by the oligomeric A*β*-mediated synapse loss and inhibition of CaMKII activation via Glu2NB-NMDAR-dependent pathway, with these processes not counterbalanced by the increased glutamate concentration (Opazo et al., 2018). Simultaneous application of AICD and A*β* led to hypoexcitability of the CA1 pyramidal neuron. Although the Glu2NB-NMDAR contribution was high, synaptic weights were reduced leading to an LTD of 60% (Figure 2, AICD & A*β*). The interplay between the upregulation of Glu2NB-NMDAR, *Ca*^2+^-dependent *K*^+^ channels *CagK*, *L*-type *Ca*^2+^ channels *CaL*, and CaMKII inhibition with synapse loss impaired LTP for high-frequency stimulation protocol.

The weak depolarization generated by 1-Hz low-frequency stimulation resulted in a weak NMDAR activation under control conditions. Synaptic weights were reduced to the minimal value of 0.6 leading to a somatic EPSP change of 40% (Figure 3, Control). AICD did not affect LTD as the hypoexcitability of the CA1 pyramidal neuron was not triggered by a low-frequency presynaptic stimulation (Figure 3, AICD).

Oligomeric A*β* led to a stronger LTD of 36% as the elevated glutamate transmission provided stronger local depolarization, while hypoexcitability was not triggered (Figure 3, **A***β*). The majority of synaptic weights reached a minimal value of 0.6. Increased levels of both AICD and oligomeric A*β* slightly strengthened LTD up to 39% due to the enhanced glutamate transmission and increased Glu2NB-NMDAR synaptic conductance (Figure 3, AICD & A*β*).

The results show the qualitative agreement with experimental findings. In control conditions, 100 pulses at 100 Hz induced 191% LTP and 500 pulses at 1 Hz led to 57% LTD in hippocampal CA1 pyramidal neurons in rats (Pousinha et al., 2017; Taylor et al., 2021). Elevated AICD concentrations prevented LTP and did not affect LTD (Pousinha et al., 2017). Oligomeric A*β* impaired LTP (Opazo et al., 2018) and strengthened LTD (Taylor et al., 2021). We modeled the joint influence of AICD and A*β*, and observed abolishment of LTP and upregulated LTD.

### 3.2 GluN2B-NMDAR blockade rescues synaptic functioning under AICD and A*β* conditions

**Figure 4:**
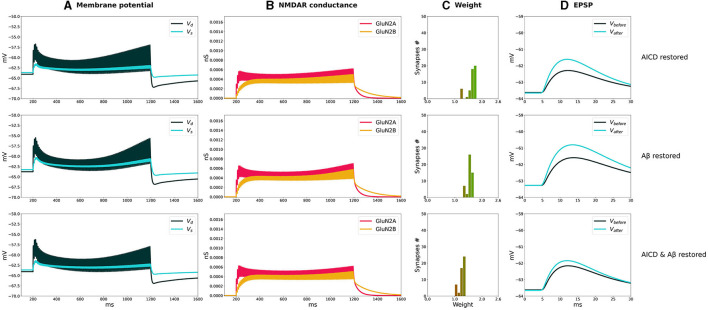
Partial blockade of Glu2NB-NMDARS restores LTP in control, AICD, A*β*, and AICD & A*β* conditions. Presynaptic inputs were stimulated at 100 Hz for 1 s. GluN2B-NMDAR synaptic conductance was increased by a factor of 4 in AICD and AICD & A*β* conditions. **(A)** Membrane potential in soma *V*_*s*_ (cyan line) and at a single synapse *V*_*d*_ (dark green line). **(B)** GluN2A-NMDAR synaptic conductance (red line) and GluN2B-NMDAR synaptic conductance (yellow line). **(C)** Synaptic weight distribution after the LTP induction protocol. **(D)** Somatic EPSP before (black line) and after (cyan line) LTP induction protocol. Rows top to bottom: (AICD restored). Partial blockade of Glu2NB-NMDAR leaving 0.25 fraction of its active elevated value leads to the EPSP change of 152%. (A*β* restored) Partial blockade of Glu2NB-NMDAR leaving 0.6 fraction of its active value leads to the EPSP change of 144%. (AICD & A*β* restored) Partial blockade of Glu2NB-NMDAR leaving 0.18 fraction of its active elevated value leads to the EPSP change of 122%.

**Figure 5:**
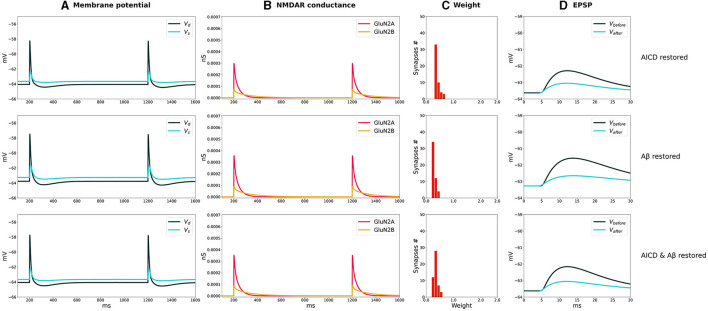
Partial blockade of Glu2NB-NMDARS normalizes LTD in control, AICD, A*β*, and AICD & A*β* conditions. Presynaptic inputs were stimulated at 1 Hz for 500 s. GluN2B-NMDAR synaptic conductance was increased by a factor of 4 in AICD and AICD & A*β* conditions. **(A)** Membrane potential in soma *V*_*s*_ (cyan line) and at a single synapse *V*_*d*_ (dark green line). **(B)** GluN2A-NMDAR synaptic conductance (red line) and GluN2B-NMDAR synaptic conductance (yellow line). **(C)** Synaptic weight distribution after the LTD induction protocol. **(D)** Somatic EPSP before (black line) and after (cyan line) LTD induction protocol. Rows top to bottom: (AICD restored) Partial blockade of Glu2NB-NMDAR leaving 0.25 fraction of its active elevated value leads to the EPSP change of 45%. (A*β* restored) Partial blockade of Glu2NB-NMDAR leaving 0.6 fraction of its active value leads to the EPSP change of 36%. (AICD & A*β* restored) Partial blockade of Glu2NB-NMDAR leaving 0.18 fraction of its active elevated value leads to the EPSP change of 40%.

We analyzed how synaptic plasticity was affected by a partial GluN2B-NMDAR blockade in the presence of increased concentrations of AICD, A*β*, for high- and low-frequency stimulations (Figures 4, 5).

In AICD conditions, Glu2NB-NMDAR synaptic conductance was increased by a factor of 4. Partial blockade of Glu2NB-NMDAR leaving 0.25 fraction of its elevated active value rescued LTP (Figure 4, AICD restored). Glu2NB-NMDAR synaptic conductance was returned to its basal value, thus preventing high *Ca*^2+^ influx, overactivation of *Ca*^2+^-dependent *K*^+^ channels, and membrane potential hyperpolarization allowing synaptic weights to reach values close to 2 and the resulting somatic EPSP increase by 152%.

An oligomeric A*β*-induced LTP impairment was restored by a partial blockade of Glu2NB-NMDAR leaving 0.6 fraction of its active value. After the stimulation, the weights increased and somatic EPSP changed by 144% as the Glu2NB-NMDAR-mediated prior overactivation of CaMKII was prevented (Figure 4, A*β* *restored*). In a similar manner, partial blockade of Glu2NB-NMDAR leaving 0.18 of its active elevated fraction rescued LTP under the conditions of increased AICD and oligomeric A*β* concentrations. Membrane potential was sufficiently depolarized and prior CaMKII overactivation was prevented, enabling synaptic weights to be strengthened and somatic EPSP to increase by 122% (Figure 4, AICD & A*β* restored).

For the low-frequency stimulation at 1 Hz, downregulation of Glu2NB-NMDAR leaving 0.25 fraction of its elevated active value did not show pronounced influence in AICD conditions. Membrane depolarization at the locations of the synapses on the dendritic branches was slightly lower to induce a weight decay, and the somatic EPSP change was equal to 45%, close to the control conditions of 40% (Figure 5, AICD restored). For the elevated oligomeric A*β* concentrations, LTD of 36% was not affected by the lower Glu2NB-NMDAR function with 0.6 fraction of its active value due to the enhanced glutamate transmission (Figure 5, A*β* restored). In the conditions of elevated AICD and oligomeric A*β* concentrations, partial blockade of Glu2NB-NMDAR synaptic conductance with 0.18 fraction of its active elevated value allowed synaptic depression to return to its normal levels of 40% (Figure 5, AICD & A*β* restored).

The results obtained align well with the experimental evidence on the Glu2NB-NMDAR function effect in synaptic plasticity in the presence of the elevated AICD and oligomeric A*β* concentrations. It was shown that ifenprodil, a specific Glu2NB-NMDAR antagonist, rescued LTP in AICD (Pousinha et al., 2017). Ifenprodil reverted oligomeric A*β*-induced inhibition of LTP (Rönicke et al., 2011). Our modeling results suggest that partial blockade of Glu2NB-NMDAR prevents impairment of LTP and LTD when both toxic peptides AICD and A*β* are present by normalizing excitability of CA1 pyramidal neuron and preventing prior CaMKII activation, thus ensuring sufficient membrane depolarization and activity of the second-messenger cascades, necessary for LTP and LTD inductions.

**Figure 6:**
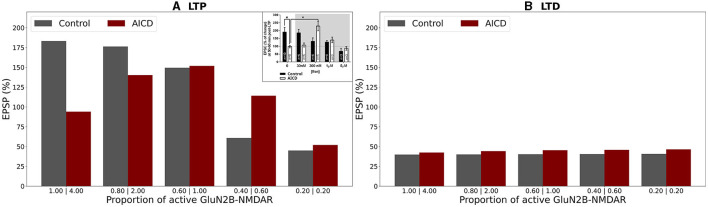
Effect of GluN2B-NMDAR blockade on somatic EPSP change in control and AICD conditions. GluN2B-NMDAR synaptic conductance was increased by a factor of 4 in AICD conditions. **(A)** LTP induction protocol. Presynaptic inputs were stimulated at 100 Hz for 1 s. Partial blockade of GluN2B-NMDAR leads to LTP impairment and prevention in control conditions (gray bars) and restores LTP in AICD conditions (red bars). Inset shows the experimental results modified from the study by Pousinha et al. (2017). Average LTP magnitude normalized to pre-LTP baseline values during 0–5 μ*M* *in vitro* bath application of idenprodil in control conditions (black bars) and in AICD conditions (white bars). Courtesy of Pousinha et al. (2017). **(B)** LTD induction protocol. Presynaptic inputs were stimulated at 1 Hz for 500 s. Blockade of GluN2B-NMDAR does not affect LTD in control conditions (gray bars) and AICD conditions (red bars).

**Figure 7:**
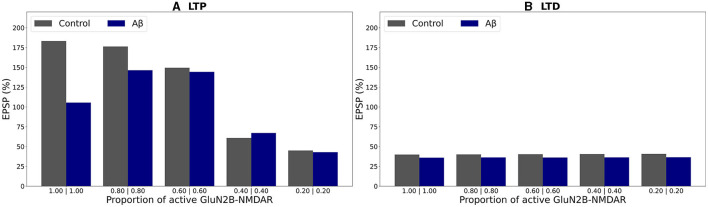
Effect of GluN2B-NMDAR blockade on somatic EPSP change in control and A*β* conditions. **(A)** LTP induction protocol. Presynaptic inputs were stimulated at 100 Hz for 1 s. Partial blockade of GluN2B-NMDAR leads to LTP impairment and prevention in control conditions (gray bars) and restores LTP in A*β* conditions (blue bars). **(B)** LTD induction protocol. Presynaptic inputs were stimulated at 1 Hz for 500 s. Blockade of GluN2B-NMDAR does not affect LTD in control conditions (gray bars) and A*β* conditions (blue bars).

**Figure 8:**
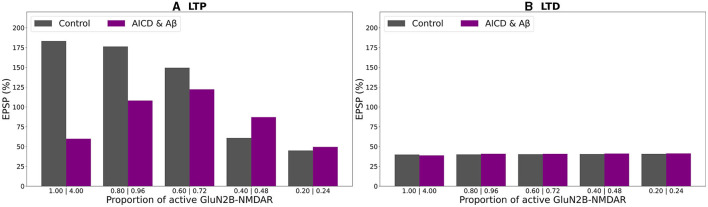
Effect of GluN2B-NMDAR blockade on somatic EPSP change in control and AICD & A*β* conditions. GluN2B-NMDAR synaptic conductance was increased by a factor of 4 in AICD & A*β* conditions. **(A)** LTP induction protocol. Presynaptic inputs were stimulated at 100 Hz for 1 s. Partial blockade of GluN2B-NMDAR leads to LTP impairment and prevention in control conditions (gray bars) and restores LTP in AICD & A*β* conditions (violet bars). **(B)** LTD induction protocol. Presynaptic inputs were stimulated at 1 Hz for 500 s. Blockade of GluN2B-NMDAR does not affect LTD in control conditions (gray bars) and AICD & A*β* conditions (violet bars).

Furthermore, we analyzed how the degree of Glu2NB-NMDAR hypofunction influences synaptic plasticity properties under both normal and AD conditions. The relative somatic EPSP changes as a function of partial Glu2NB-NMDAR blockade for elevated AICD, A*β*, and AICD & A*β* concentrations versus healthy control state are shown in Figures 6–**8**. The proportion of active GluN2B-NMDAR was calculated in relation to the maximum GluN2B-NMDAR conductance under normal conditions and was converted to the active fraction by normalizing it by the maximum GluN2B-NMDAR conductance in AICD, A*β*, and AICD & A*β* conditions, respectively. Maximum GluN2B-NMDAR conductance was increased by a factor of 4 to account for AICD and AICD & A*β* effect, while it was maintained the same for the A*β* conditions.

In the absence of AD peptides, the LTP induction protocol led to reduced somatic EPSP from 183% to 45% as the hypofunction in GluN2B-NMDAR increased, i.e., as the proportion of active GluN2B-NMDAR was lowered from 1 to 0.2 (Figure 6A, gray columns). Under AICD conditions, the bell-shape effect of partial GluN2B-NMDAR blockade was observed, where the optimal active proportion corresponded to the strongest LTP for high frequency stimulation protocol (Figure 6A, red columns). Specifically, partial blockade of the upregulated GluN2B-NMDAR channel and restoration of its active proportion to 1 instead of 4 (i.e., maintaining 0.25 fraction of its active elevated value) resulted in LTP of 152%. The proportions 2 and 0.6 (fractions 0.5 and 0.15) led to a weaker LTP of 140% and 114%, respectively, while the absence of GluN2B-NMDAR blockade or almost full inhibition of GluN2B-NMDAR (proportions 4 and 0.2, fractions 1 and 0.05) converted LTP to LTD of 94% and 45%, respectively. Partial blockade of GluN2B-NMDAR left LTD unaffected at 39%–41% in control conditions (Figure 6B, gray columns). The impairment of GluN2B-NMDAR functioning did not show such a strong non-linear effect on LTD leaving the EPSP change at 42%–46% (Figure 6B, red columns).

The modeling study effectively replicated the experimental findings on the impact of the GluN2B-NMDAR inhibitor ifenprodil on synaptic plasticity (Figure 6A inset; Pousinha et al., 2017). In control conditions, increasing ifenprodil concentration up to 1 μ*M* reduced LTP, and 5 μ*M* converted 190% LTP to 75% LTD for high-frequency stimulation. Ifenprodil application did not affect LTD for low-frequency stimulation. In AICD conditions, a bell-shape effect was observed for LTP induction protocol, and 300 *nM* of ifenprodil rescued LTP.

In the presence of oligomeric A*β* forms, optimal GluN2B-NMDAR blockade maintaining a proportion of 0.6 (or a fraction 0.6 of its active not-elevated value) restored LTP to 144% if compared to 105% for the fully functional GluN2B-NMDAR and high-frequency stimulation (Figure 7A, blue bars). Increased hypofunction of GluN2B-NMDAR led to the induction of LTD. The blockage of this receptor did not alter LTD for low frequency stimulation protocol (Figure 7B, blue bars). The results are consistent with the experimental work (Opazo et al., 2018) which demonstrated that oligomeric A*β* promoted CaMKII pre-activation and impairment of LTP. Blockade of GluN2B-NMDAR by ipenfrodil prevented CaMKII activation, and CaMKII inhibition was sufficient to rescue LTP.

In a similar manner, alterations in somatic EPSP appeared as a bell-shape curve, dependent on GluN2B-NMDAR function, when the concentrations of both AICD and A*β* were elevated (Figure 8A, violet bars). Optimal GluN2B-NMDAR blockade with 0.72 proportion (0.18 fraction of its active elevated value) restored LTP to 122 %, while the higher of lower levels of its activity failed to strengthen the synapses sufficiently. For low-frequency stimulation, LTD was not affected by the GluN2B-NMDAR hypofunction (Figure 8B, violet bars).

**Figure 9:**
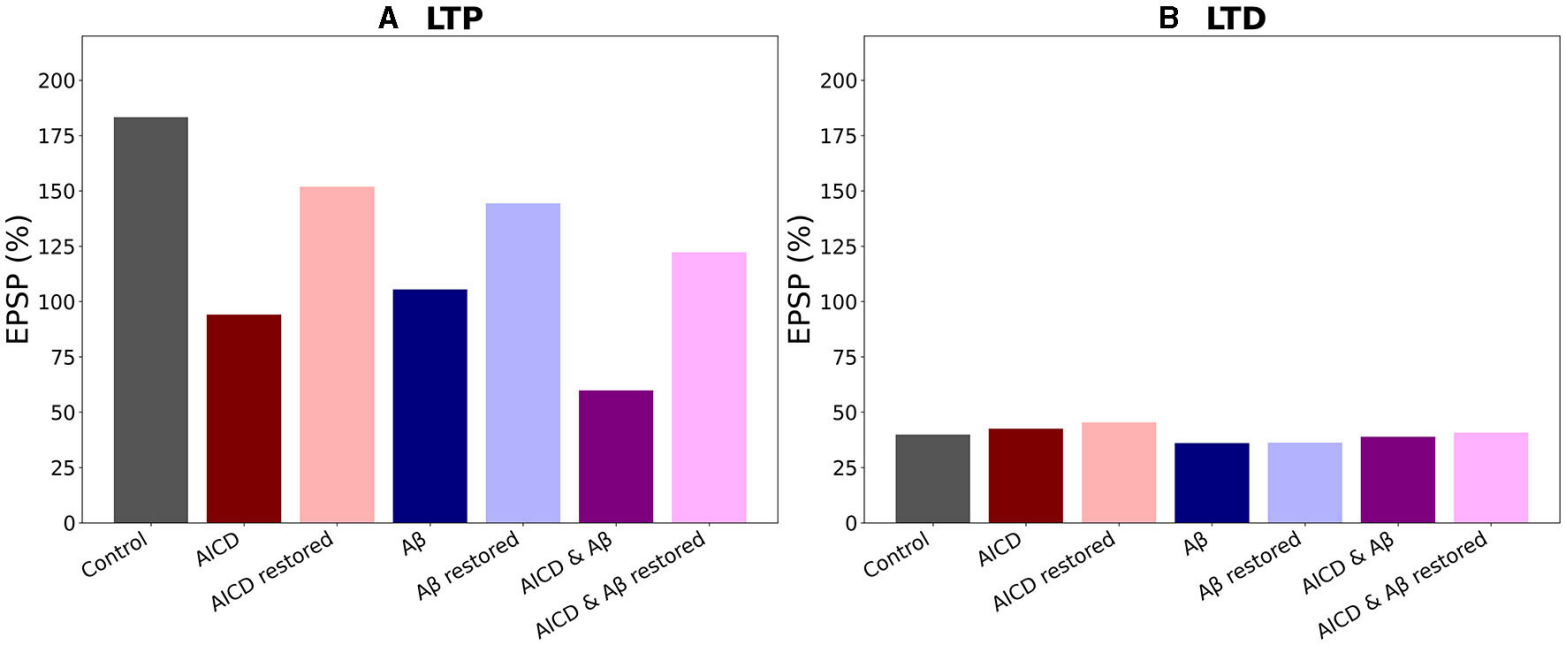
Optimal blockade of GluN2B-NMDAR restores synaptic functionality. **(A)** Somatic EPSP change for LTP induction protocol. GluN2B-NMDAR synaptic conductance was increased by a factor of 4 in AICD conditions and AICD conditions & A*β* conditions. Left to right columns: (Control) Somatic EPSP change is 183% (gray bar); (AICD) Somatic EPSP change is 94% (red bar); (AICD restored) Active GluN2B-NMDAR proportion is 1 of its elevated value of 4, i.e., the active fraction is 0.25; somatic EPSP change is 152% (light red bar); (A*β*) Somatic EPSP change is 105% (blue bar); (A*β* restored) Active GluN2B-NMDAR proportion is 0.6; somatic EPSP change is 144% (light blue bar); (AICD & A*β*) Somatic EPSP change is 60% (violet bar); (AICD & A*β* restored) Active GluN2B-NMDAR proportion is 0.72 of its elevated value of 4, i.e., the active fraction is 0.18; somatic EPSP change is 122% (pink bar). **(B)** Somatic EPSP change for LTD induction protocol. Left to right columns: (Control) Somatic EPSP change is 40% (gray bar); (AICD) Somatic EPSP change is 42% (red bar); (AICD restored) Active GluN2B-NMDAR proportion is 1 of its elevated value of 4, i.e., the active fraction is 0.25; somatic EPSP change is 45% (light red bar); (A*β*) Somatic EPSP change is 36% (blue bar); (A*β* restored) Active GluN2B-NMDAR proportion is 0.6; somatic EPSP change is 36% (light blue bar); (AICD & A*β*) Somatic EPSP change is 39% (violet bar); (AICD & A*β* restored) Active GluN2B-NMDAR proportion is 0.72 of its elevated value of 4, i.e., the active fraction is 0.18; somatic EPSP change is 40% (pink bar).

Summary of the results is presented in Figure 9. In control conditions, LTP reached 183% for high frequency stimulation (Figure 9A, gray bar). Increased AICD levels led to a slight LTD of 94%, and GluN2B-NMDAR blockade to its active initial proportion 1 restored LTP to 152% (Figure 9A, red and light red bars). Oligomeric A*β* impaired LTP to 105% that was restored to 144% by the GluN2B-NMDAR blockade leaving 0.6 of its active proportion (Figure 9A, blue and light blue bars). For the elevated concentrations of both AICD and A*β*, somatic EPSP LTD to 60% was prevented and converted to LTP of 122% by the GluN2B-NMDAR blockade leading to 0.72 of its active proportion (Figure 9A, violet and pink bars).

Taken together, the results obtained in this study indicated that optimal antagonism of GluN2B-NMDAR allowed to restore synaptic functionality in AD through normalizing intrinsic excitability and molecular pathways, responsible for LTP induction.

## 4 Discussion

We extended the Glu2NB-NMDAR and Glu2NA-NMDAR subunit-dependent voltage-based model of synaptic plasticity at hippocampal CA3-CA1 synapses (Dainauskas et al., 2023) to analyse the effects of elevated levels of two AID peptides, AICD and oligomeric A*β*, and modeled synaptic modifications in a cluster of synapses distributed on the proximal apical dendrites of a detailed compartmental model of a CA1 pyramidal neuron. We optimized the ion channels of the CA1 pyramidal neuron model to account for reduced intrinsic excitability in AICD conditions (Pousinha et al., 2019). We validated the models against the experimental data of synaptic plasticity impairment under the AICD and oligomeric A*β* conditions and in the presence of GluN2B-NMDAR blockade (Pousinha et al., 2017, 2019; Opazo et al., 2018). We confirmed that GluN2B-NMDAR dysfunction leads to LTP impairment and transformation to LTD in control conditions (Pousinha et al., 2017). We successfully replicated the bell-shape effect of GluN2B-NMDAR downregulation in the presence of the elevated AICD concentration for high-frequency stimulation and LTP rescue by the optimal blockade of GluN2B-NMDAR (Pousinha et al., 2017). We qualitatively reproduced experimental findings that GluN2B-NMDAR downregulation restores LTP in oligomeric A*β* conditions. Increased AICD levels enhanced intracellular *Ca*^2+^ concentration via GluN2B-NMDAR and *L*-type *Ca*^2+^ channels, which activated the nearby *Ca*^2+^-dependent *K*^+^ channels and led to hypoexcitability of a CA1 pyramidal neuron. Oligomeric forms of A*β* caused synapse loss and prevented CaMKII activation via Glu2NB-NMDAR-dependent pathway. Optimal blockade of GluN2B-NMDAR normalized *Ca*^2+^-dependent *K*^+^ channel functioning, restored excitability and allowed CaMKII to be phoshorylated to induce LTP.

We investigated the effect of simultaneous application of AICD and A*β* and showed that AICD-triggered hypoexcitability and A*β*-induced prevention of CaMKII activation can be averted by downregulation of Glu2NB-NMDAR functioning. Our modeling studies predict that AICD & A*β* -mediated impairment in LTP and LTD can be reversed by the optimal GluN2B-NMDAR antagonism. We demonstrated in a mechanistic way that impairment in synaptic plasticity in AD conditions depends on the interplay between upregulation of Glu2NB-NMDAR, *Ca*^2+^-dependent *K*^+^ channels *CagK*, *L*-type *Ca*^2+^ channels *CaL*, CaMKII inhibition, and synapse loss.

We showed that a reduction of Glu2NB-NMDAR conductance is sufficient to restore synaptic plasticity in AICD, A*β*, and AICD & A*β* conditions, despite the fact that ion channels, neurotransmitter release, and spine density were also affected by AD. Moreover, our model makes the experimentally testable prediction that lower or higher values of active Glu2NB-NMDAR conductance cannot result in LTP rescue. For AICD conditions, this optimal active fraction of Glu2NB-NMDAR corresponded to the initial value of Glu2NB-NMDAR in control condition. Reduced activity of the overexpressed Glu2NB-NMDAR prevented upregulation of *Ca*^2+^-dependent *K*^+^ channels *CagK*, restored excitability of CA1 pyramidal neuron, and led to the rescue of LTP. The model suggested that smaller values of active Glu2NB-NMDAR were not sufficient to trigger the LTP induction pathways, while large values resulted in the overactivation of *CagK* channels, insufficient membrane potential depolarization, and impaired LTP. For A*β* and AICD & A*β* conditions, the optimal active fraction of Glu2NB-NMDAR was lower than in control conditions. For increased oligomeric A*β* concentrations, the optimal active fraction of Glu2NB-NMDAR was equal to 0.6 if compared to the control conditions. The reason behind this effect was that normal functioning of Glu2NB-NMDAR overactivated CaMKII, the main protein in the LTP molecular pathway, and prevented LTP induction. Small active fraction of Glu2NB-NMDAR failed to sufficiently activate CaMKII and trigger LTP. For AICD & A*β* conditions, the optimal active fraction of Glu2NB-NMDAR was equal to 0.72 of its active value, if compared to the control conditions. In this case, block of Glu2NB-NMDAR overexpression prevented both upregulation of *Ca*^2+^-dependent *K*^+^ channels *CagK* and overactivation of CaMKII, and rescued LTP.

The modeling study links complex interactions among Glu2NB-NMDAR, excitability of CA1 pyramidal neuron, and LTP induction pathways, and allows to get insights into the mechanisms of LTP impairment and its rescue.

Our modeling study enables to analyse these processes and allows prediction of the joint effect of AD peptides and the influence of partial GluN2B-NMDAR blockade to restore the properties of synaptic plasticity.

Computational modeling offers a powerful technique to interpret the mechanisms of AD, including its pathophysiology, neurobiology, and potential interventions, and allows the integration of experimental findings at many different scales, from genes, molecules, and synapses up to neurons, networks, and the whole brain. Computational models of AD can be categorized into molecular and sub-cellular, single cell, neural circuits, and large-scale brain network models. Molecular and subcellular models of AD pathophysiology focus on detailed description of complex biochemical interactions of APP processing, A*β*, tau, tangles, inflammation, intracellular *Ca*^2+^ concentration, and a network of proteins and phosphatases responsible for LTP and LTD induction (Pallitto and Murphy, 2001; Proctor and Gray, 2010; Anastasio, 2011, 2013, 2014; Schmidt et al., 2012; De Caluwé and Dupont, 2013; Kyrtsos and Baras, 2013; Ortega et al., 2013; Proctor et al., 2013). At a single neuron level, detailed biophysical models of CA1 pyramidal and cortical neurons were employed to analyse the influence of A*β* on neuronal excitability (Morse et al., 2010; Culmone and Migliore, 2012; Romani et al., 2013). Neural circuit models of AD is another increasingly expanding class of studies representing specific brain networks affected by the disease, and typically focus on the altered synaptic function, activity of individual neurons, excitatory-inhibitory balance, and oscillations (Hasselmo, 1997; Bhattacharya et al., 2011; Zou et al., 2011; Abuhassan et al., 2012; Bianchi et al., 2014; Rowan et al., 2014; Bachmann et al., 2020; Li et al., 2020, 2023; Ness and Schultz, 2021). Large-scale brain network models simulate the development, manifestation, and progression of AD at a macroscopic level of brain regions connecting mean field models of neural activity to multimodal EEG, MEG, and fMRI data (Haan et al., 2017). Recently, a whole-brain simulation neuroinformatics platform The Virtual Brain (www.thevirtualbrain.org) has been developed, driven by the neural mass modeling approach and multi-modal structural, functional (MRI, diffusion MRI, and PET), neurophysiological (EEG and MEG), genetic, molecular, and cognitive data (Ritter et al., 2013; Spiegler and Jirsa, 2013; Zimmermann et al., 2018; Stefanovski et al., 2019, 2021).

Our modeling study falls into a class of single neuron models. Previous studies of hippocampal CA1 neuron properties in AD analyzed the effect of A*β* on the intrinsic excitability and short-term plasticity, influence of CREB on pattern storage and recall (Culmone and Migliore, 2012; Romani et al., 2013; Bianchi et al., 2014). At a single neuron level, Culmone and Migliore (2012) investigated the progressive effect of A*β* accumulation on membrane properties of hippocampal CA1 pyramidal neuron and showed that A*β*-mediated reduction of *K*_*A*_, *K*_*DR*_, and *Na*^+^ peak conductances in the sections of dendritic tree and decrease in synaptic conductances led to the decreased firing probability. Although basing the model on experimental data obtained with very high concentrations of A*β* (100 μ*M*). The modeling study predicted that increase of *K*_*A*_ or *Na*^+^ currents combined with a similar increase in synaptic conductance may restore spike probability of a CA1 neuron. Romani et al. (2013) implemented the A*β*-induced enhancement in the initial release probability at the CA3-CA1 synapses of the hippocampus and demonstrated that the altered synaptic short-term plasticity of the synapse favored synaptic depression over facilitation, and significantly modified synaptic integration properties. The potential pharmacological treatment was suggested as the increase in *K*_*A*_, *Na*^+^ conductances, and enhancement of AMPAR synaptic conductance as a result of CREB activity boosting.

We extended the studies on CA1 pyramidal neurons and analyzed the joint effect of AICD and A*β* on intrinsic excitability, and in particular, on synaptic plasticity in AD. We integrated the experimental findings on AICD and A*β* and presented prediction of the complex interaction effects of AD peptides and the influence of partial GluN2B-NMDAR blockade to normalize the properties of synaptic plasticity. The novelty of our study is the demonstration that modulation of GluN2B-NMDAR activity by its partial blockade has a potential to prevent A*β*—mediated synaptotoxicity and AICD-induced hypoexcitability, and restore synaptic plasticity in early AD. We aim to extend our study to modeling the storage and recall in hippocampal CA1 network under the influence of elevated concentrations of AD peptides. Hippocampal synaptic plasticity plays a critical role in memory formation and retrieval; therefore, mechanistic framework of complex processes facilitates understanding of cognitive impairment in neurodegenerative diseases and suggests potential targets to preserve and maintain brain network functionality.

## Data availability statement

The datasets presented in this study can be found in online repositories. The names of the repository/repositories and accession number(s) can be found in the article/Supplementary material.

## Author contributions

JD: Data curation, Formal analysis, Investigation, Methodology, Software, Visualization, Writing – review & editing. PV: Data curation, Investigation, Methodology, Software, Writing – review & editing. SM: Conceptualization, Investigation, Writing – review & editing. HM: Conceptualization, Data curation, Funding acquisition, Investigation, Methodology, Supervision, Writing – review & editing. MM: Conceptualization, Funding acquisition, Supervision, Writing – review & editing. AS: Conceptualization, Funding acquisition, Investigation, Methodology, Project administration, Resources, Supervision, Validation, Writing – original draft, Writing – review & editing.
