# Supplementary material for: Altered synaptic plasticity at hippocampal CA1–CA3 synapses in Alzheimer's disease: integration of amyloid precursor protein intracellular domain and amyloid beta effects into computational models
Source: Front Comput Neurosci. 2023 Dec 7;17:1305169. doi: 10.3389/fncom.2023.1305169 (PMC10733499; doi:10.3389/fncom.2023.1305169)
Supplement: Supplementary file 1 [file Data_Sheet_1.pdf]

Supplementary Material

Altered synaptic plasticity at hippocampal CA1-CA3 synapses in Alzheimer's disease: integration of amyloid precursor protein intracellular domain and amyloid beta effects into computational models

Justinas J. Dainauskas [1,2], Paola Vitale [3], Sebastien Moreno [4], Hélène Marie [4], Michele Migliore [3], and Ausra Saudargiene [1,2,*]

[1]Neuroscience Institute. Lithuanian University of Health Sciences, Kaunas, Lithuania
[2]Department of Informatics, Vytautas Magnus University, Kaunas, Lithuania
[3]Institute of Biophysics, National Research Council, Palermo, Italy
[4]Université Côte d'Azur, Centre National de la Recherche Scientifique (CNRS), Institut de Pharmacologie Moléculaire et Cellulaire (IPMC), Valbonne, France

Corresponding author: ausra.saudargiene@lsmu.lt

1 SUPPLEMENTARY DATA

We extended a phenomenological voltage-dependent NMDAR-based synaptic plasticity model, developed in our previous study (Dainauskas et al., 2023), and incorporated the effects of amyloid precursor protein intracellular domain (AICD) and amyloid beta (Aβ) on synaptic plasticity in Alzheimer's disease (AD). We studied the influence of partial GluN2B-NMDAR blockade on synaptic modifications at a cluster of CA3-CA1 synapses distributed randomly onto apical dendrites of a CA1 neuron.

We used a morphology reconstruction of a CA1 pyramidal neuron downloaded from http://www.neuromorpho.org (Peng et al., 2016) (cell $fx\_CA1\_7.CNG.swc$). The model was implemented with channel kinetics used in (Migliore et al., 2018) (ModelDB accession number 244688). We optimized the model to reproduce the recordings under the AICD conditions (Pousinha et al., 2019). Under the Aβ conditions, the excitability profile of CA1 pyramidal neuron was not altered.

Frequency-dependent synaptic plasticity induction protocol (Pousinha et al., 2017) was used: presynaptic input was stimulated using a conditioning protocol that consisted of 100 pulses at 100 Hz (LTP protocol) or 500 pulses at 1 Hz (LTD protocol). Synaptic placticity outcome was measured as the change in the somatic excitatory postsynaptic potential (EPSP). To estimate the EPSP change, a presynaptic stimulus was delivered before and after the conditioning stimulation, and the resulting ratio between the maximal values of the resulting EPSPs in soma was calculated.

1.1 NMDAR-based voltage-dependent synaptic plasticity model

A voltage-based model of synaptic plasticity consists of LTD and LTP components and includes the effect of postsynaptic Glu2NA-NMDAR and Glu2NB-NMDAR subunits (Dainauskas et al., 2023). We modified the LTP component to account for the influence of Aβ in preventing CaMKII activation in a Glu2NB-NMDAR-dependent manner.

The instantaneous weight change $\frac{d}{dt}w_{AMPA}(t)$ consists of two additive NMDAR-dependent LTP and LTD contributions, $\frac{d}{dt}w_{LTP}(t)$ and $\frac{d}{dt}w_{LTD}(t)$ (Dainauskas et al., 2023):

$$\frac{d}{dt}w_{AMPA}(t) = \frac{d}{dt}w_{LTP}(t)\left(w_{max} - w(t)\right) - \frac{d}{dt}w_{LTD}(t)\left(w(t) - w_{min}\right), \tag{S1}$$

where w_{max} and w_{min} set the limits for synaptic weight w_{AMPA}. The LTP component $\frac{d}{dt}w_{LTP}(t)$ is expressed as the product of the NMDAR-dependent function $\phi_{NMDA_{LTP}}(t)$ and a low-filtered membrane potential $\overline{V}_{LTP}(t)$ (Dainauskas et al., 2023):

$$\frac{d}{dt}w_{LTP}(t) = A_{LTP}\ \phi_{NMDA_{LTP}}(t)\ \overline{V}_{LTP}(t), \tag{S2}$$

where A_{LTP} is the LTP amplitude parameter. Similarly, the LTD component $\frac{d}{dt}w_{LTD}(t)$ is proportional to the product of the NMDAR-dependent function $\phi_{NMDA_{LTD}}(t)$ and a low-filtered membrane potential $\overline{V}_{LTD}(t)$ (Dainauskas et al., 2023):

$$\frac{d}{dt}w_{LTD}(t) = A_{LTD}\ \phi_{NMDA_{LTD}}(t)\ \overline{V}_{LTD}(t)\ \overline{X}(t), \tag{S3}$$

where $\overline{X}(t)$ is a presynaptic activity variable, and A_{LTD} is the LTD amplitude parameter.

Function $\phi_{NMDA_{LTP}}(t)$ governs LTP induction and is caused by the filtered NMDAR conductance $\overline{g}_{NMDA_{LTP}}(t)$. Function $\phi_{NMDA_{LTD}}(t)$ accounts for LTD, and is triggered by the filtered NMDAR conductance $\overline{g}_{NMDA_{LTD}}(t)$.

In this work, the LTP function $\phi_{NMDA_{LTP}}(t)$ is modified and described as difference of two Hill functions, leading to a bell-shaped curve to capture the influence of Aβ:

$$\phi_{NMDA_{LTP}}(t) = \frac{1}{1+\left(\frac{K_{a1_{LTP}}}{\overline{g}_{NMDA_{LTP}}(t)}\right)^{n_{LTP}}} - \frac{1}{1+\left(\frac{K_{a2_{LTP}}}{\overline{g}_{NMDA_{LTP}}(t)}\right)^{n_{LTP}}} - \theta_{LTP}(t), \tag{S4}$$

and the LTP function $\phi_{NMDA_{LTP}}(t)$ is equal (Dainauskas et al., 2023):

$$\phi_{NMDA_{LTD}}(t) = \frac{1}{1+\left(\frac{K_{aLTD}}{\overline{g}_{NMDA_{LTD}}(t)}\right)^{n_{LTD}}} - \theta_{LTD}(t), \tag{S5}$$

where $\overline{g}_{NMDA_{LTP}}(t)$ and $\overline{g}_{NMDA_{LTD}}(t)$ are the filtered NMDAR conductances, n_{LTP} and n_{LTD} are the Hill coefficients, θ_{LTP} and θ_{LTD} are the moving thresholds of $\phi_{NMDA_{LTP}}(t)$ and $\phi_{NMDA_{LTD}}(t)$ for for LTP and LTD induction. LTP function $\phi_{NMDA_{LTP}}(t)$ is proportional to the difference of two Hill-type functions with the values $K_{a1_{LTP}}$ and $K_{a2_{LTP}}$ of $\overline{g}_{NMDA_{LTP}}(t)$, producing half of their activation. Coefficient $K_{a1_{LTP}}$ is lower than $K_{a2_{LTP}}$, leading $\phi_{NMDA_{LTP}}(t)$ to a bell-shaped function.

The moving threshold $\theta_{LTP}(t)$ lowers the activity of the LTP function $\phi_{NMDA_{LTP}}(t)$ and depends on the increased LTD function $\phi_{NMDA_{LTD}}(t)$ (Dainauskas et al., 2023):

$$\tau_{\theta_{LTP}}\frac{d}{dt}\theta_{LTP}(t) = -\ \theta_{LTP}(t) + \phi_{NMDA_{LTD}}(t), \tag{S6}$$

where $\tau_{\theta_{LTP}}$ is a time constant.

Similarly, the moving threshold $\theta_{LTD}(t)$ is increased by the LTP function $\phi_{NMDA_{LTP}}(t)$ and leads to the reduced activity of the LTD function $\phi_{NMDA_{LTD}}(t)$ (Dainauskas et al., 2023):

$$\tau_{\theta_{LTD}}\frac{d}{dt}\theta_{LTD}(t) = -\ \theta_{LTD}(t) + \phi_{NMDA_{LTP}}(t), \tag{S7}$$

where $\tau_{\theta_{LTD}}$ is a time constant.

The moving threshold $\theta_{LTD}(t)$ is increasing, if $\phi_{NMDA_{LTP}}(t)$ is strongly activated and LTP is induced, thus vetoing LTD. The LTP threshold $\theta_{LTP}(t)$ may also increase if the LTD function $\phi_{NMDA_{LTD}}(t)$ accumulates, leading to LTD. Thus, the moving thresholds implement competition between LTP and LTD.

The filtered NMDAR-dependent variables for LTP and LTD components are described (Dainauskas et al., 2023):

$$\tau_{NMDA_{LTP}} \frac{d}{dt}\overline{g}_{NMDA_{LTP}}(t) = -\,\overline{g}_{NMDA_{LTP}}(t) + g_{NMDA_{LTP}}(t), \tag{S8}$$

$$\tau_{NMDA_{LTD}} \frac{d}{dt}\overline{g}_{NMDA_{LTD}}(t) = -\,\overline{g}_{NMDA_{LTD}}(t) + g_{NMDA_{LTD}}(t), \tag{S9}$$

where $\tau_{NMDA_{LTP}}$, $\tau_{NMDA_{LTD}}$ are the time constants, and $g_{NMDA_{LTP}}(t)$, $g_{NMDA_{LTD}}(t)$ are conductances of postsynaptic NMDAR that incorporate both GluN2A-NMDAR and GluN2B-NMDAR subunits with a different weighting coefficient k_{LTP} and k_{LTD} for LTP and LTD components, respectively (Dainauskas et al., 2023):

$$g_{NMDA_{LTP}}(t) = k_{LTP}\, g_{NMDA_{GluN2B}}(t) + (1 - k_{LTP})\, g_{NMDA_{GluN2A}}(t) \tag{S10}$$

$$g_{NMDA_{LTD}}(t) = k_{LTD}\, g_{NMDA_{GluN2B}}(t) + (1 - k_{LTD})\, g_{NMDA_{GluN2A}}(t) \tag{S11}$$

Following (Morishita et al., 2007; Pousinha et al., 2017; Andrade-Talavera et al., 2016) we assume that LTP is mainly governed by GluN2B-NMDAR subunit, and LTD is mediated by GluN2A-NMDAR (or other) subunit. We set the coefficient of GluN2B-NMDAR effect on LTP $k_{LTP} = 0.8$, and coefficient of GluN2B-NMDAR effect on LTD $k_{LTD} = 0.2$

Variables $\overline{V}_{LTP}(t)$ and $\overline{V}_{LTD}(t)$ are the functions of the filtered membrane potential $V(t)$ at the synapse location contributing to the LTP and LTD components and are expressed (Dainauskas et al., 2023):

$$\tau_{LTP} \frac{d}{dt}\overline{V}_{LTP}(t) = -\,\overline{V}_{LTP}(t) + \left[V(t) - \theta_{V_{LTP}}\right]_+, \tag{S12}$$

$$\tau_{LTD} \frac{d}{dt}\overline{V}_{LTD}(t) = -\,\overline{V}_{LTD}(t) + \left[V(t) - \theta_{V_{LTD}}\right]_+, \tag{S13}$$

where τ_{LTP}, τ_{LTD} are the time constants and $\theta_{V_{LTP}}$, $\theta_{V_{LTD}}$ are the thresholds for the LTP and LTD components.

A presynaptic activity variable $\overline{X}$ in Eq. S3 is calculated as a low pass filter of the presynaptic spike train $\Sigma_i \delta(t - t_i)$ with time constant τ_δ using $\tau_\delta \frac{d}{dt}\overline{X}(t) = -\overline{X}(t) + \Sigma_i \delta(t - t_i)$.

Parameters of synaptic plasticity model are given in Table S1.

Parameters of synaptic plasticity model were adjusted manually following the model assumptions and conditions under which the main functions $\phi_{NMDA_{LTP}}(t)$, $\phi_{NMDA_{LTD}}(t)$, $\overline{g}_{NMDA_{LTP}}(t)$, $\overline{g}_{NMDA_{LTD}}(t)$ (Eq. S4, S5, S8, S9) have to be activated.

Specifically, LTP function $\phi_{NMDA_{LTP}}(t)$ is triggered by $\overline{g}_{NMDA_{LTP}}(t)$, that depends mainly on Glu2NB-NMDAR synaptic conductance function $g_{NMDA_{Glu2NB}}(t)$, as it is assumed that Glu2NB-NMDAR mediates LTP. Therefore, its activation parameter $K_{a1_{LTP}}$ is adjusted so that $\phi_{NMDA_{LTP}}(t)$ is weak for low frequency (1 Hz) stimulation and becomes strong for high frequency (100 Hz) stimulation. In a similar manner, LTD function $\phi_{NMDA_{LTD}}(t)$ is induced by $\overline{g}_{NMDA_{LTD}}(t)$, that is dependent mainly on Glu2NA-NMDAR synaptic conductance $g_{NMDA_{Glu2NA}}(t)$, as Glu2NA-NMDAR is responsible for LTD. Its activation parameter $K_{a_{LTD}}$ is adjusted so that low frequency stimulation triggers $\phi_{NMDA_{LTD}}(t)$. If the frequency of stimulation increases, $\phi_{NMDA_{LTD}}(t)$ becomes inhibited by $\phi_{NMDA_{LTD}}(t)$.

LTP function $\phi_{NMDA_{LTP}}(t)$ has a larger Hill coefficient n_{LTP} if compared to Hill coefficient n_{LTD} of LTD function $\phi_{NMDA_{LTD}}(t)$ to ensure steeper activation profile of LTP component for high frequency stimulation. Time constant of $\overline{g}_{NMDA_{LTP}}(t)$ is large to represent the time course of CaMKII activity. Time constant of $\overline{g}_{NMDA_{LTD}}(t)$ is shorter to approximate the activity of LTD induction pathways.

1.2 Synaptic conductances of GluN2A-NMDAR and GluN2B-NMDAR subunits

Synaptic conductances of GluN2A-NMDAR and GluN2B-NMDAR subunits are modeled following (Destexhe et al., 1994).

Presynaptic activation was modeled as a brief pulse of glutamate concentration (1 mM during 1 ms) that triggered binding of the transmitter to AMPAR and NMDAR, and induced transition of receptors from closed to open states.

$$g_{NMDA_{[GluN2\dagger]}}(t) = f_{Mg}(R_{on_{[GluN2\dagger]}} - R_{off_{[GluN2\dagger]}})K_{[GluN2\dagger]}, \tag{S14}$$

where $[GluN2\dagger]$ denotes two types of NMDAR GluN2 subunits, GluN2A-NMDAR and GluN2B-NMDAR, $R_{on_{[GluN2\dagger]}}$ and $R_{off_{[GluN2\dagger]}}$ are the fraction of open and closed GluN2A-NMDAR and GluNB-NMDAR, $K_{[GluN2\dagger]}$ is the scaling coefficient of GluN2A-NMDAR and GluN2B-NMDAR conductances, and $f_{[Mg^{2+},V(t)]}$ is a NMDAR gating function, dependent of extracellular magnesium concentration $[Mg^{2+}]$ and local membrane potential $V(t)$:

$$f_{[Mg^{2+},V(t)]} = \frac{1}{(1+e^{-0.062V(t)})([Mg^{2+}]/3.57)}. \tag{S15}$$

$R_{on_{[GluN2\dagger]}}$, $R_{off_{[GluN2\dagger]}}$ and $R_{inf_{[GluN2\dagger]}}$ of GluN2A-NMDAR and GluNB-NMDAR are equal:

$$\tau_{[GluN2\dagger]}\frac{d}{dt}R_{on_{[GluN2\dagger]}} = R_{inf_{[GluN2\dagger]}} - R_{on_{[GluN2\dagger]}}, \tag{S16}$$

$$\frac{d}{dt}R_{off_{[GluN2\dagger]}} = -\beta_{[GluN2\dagger]}R_{off_{[GluN2\dagger]}}, \tag{S17}$$

and

$$R_{inf_{[GluN2\dagger]}} = \frac{\alpha_{[GluN2\dagger]}\,Glu}{\alpha_{[GluN2\dagger]}\,Glu + \beta_{[GluN2\dagger]}}, \tag{S18}$$

where $\alpha_{[GluN2\dagger]}$ and $\beta_{[GluN2\dagger]}$ are forward and backward binding rates are of GluN2A-NMDAR and GluNB-NMDAR, adjusted following (Cull-Candy et al., 2001), and Glu is the maximal concentration of glutamate during the synapse activation..

Time constant $\tau_{[GluN2\dagger]}$ is defined:

$$\tau_{[GluN2\dagger]} = \frac{1}{\alpha_{[GluN2\dagger]}\,Glu + \beta_{[GluN2\dagger]}}. \tag{S19}$$

GluN2B-NMDAR subunit has a slower inactivation time than GluN2A-NMDAR subunit. Kinetic parameters of forward and backward binding rates are adjusted (Cull-Candy et al., 2001).

Current through NMDAR gated channel consists of sodium $I_{Na,NMDA}$ and calcium $I_{Ca,NMDA}$ currents $I_{NMDA} = I_{Na,NMDA} + I_{Ca,NMDA}$ that are expressed:

$$I_{Na,NMDA}(t) = 0.94 \times g_{NMDA}(t)(V_d - E_{NMDA}), \tag{S20}$$

$$I_{Ca,NMDA}(t) = 0.06 \times g_{NMDA}(t)(V_d - E_{NMDA}), \tag{S21}$$

$$g_{NMDA}(t) = (g_{NMDA_{[GluN2A]}}(t) + g_{NMDA_{[GluN2B]}}(t))\hat{g}_{NMDA}, \tag{S22}$$

where $\hat{g}_{NMDA}$ is the maximal conductance of NMDAR.

1.3 Synaptic conductance of AMPAR

The non-specific current through the AMPAR gated channel is:

$$I_{AMPA}(t) = w_{AMPA}(t)\, g_{AMPA}(t)(V_d - E_{AMPA}), \tag{S23}$$

where $w(t)$ is a synaptic weight defined by Eq. S1.

Presynaptic activation was modeled as a brief pulse of glutamate concentration (1 mM during 1 ms) that triggered binding of the transmitter to AMPAR.

AMPAR synaptic conductance is described (Destexhe et al., 1994):

$$g_{AMPA}(t) = (R_{on_{AMPA}} - R_{off_{AMPA}})\hat{g}_{AMPA}, \tag{S24}$$

where $R_{on_{AMPA}}$ and $R_{off_{AMPA}}$ are the fraction of open and closed AMPAR, $\hat{g}_{AMPA}$ is the maximal AMPAR conductance.

$R_{on_{AMPA}}$, $R_{off_{AMPA}}$ are equal:

$$\tau_{AMPA}\frac{d}{dt}R_{on_{AMPA}} = R_{inf_{AMPA}} - R_{on_{AMPA}}, \tag{S25}$$

$$\frac{d}{dt}R_{off_{AMPA}} = -\beta_{AMPA}R_{off_{AMPA}}, \tag{S26}$$

and $R_{inf_{AMPA}}$ is expressed:

$$R_{inf_{AMPA}} = \frac{\alpha_{AMPA}\, Glu}{\alpha_{AMPA}\, Glu + \beta_{AMPA}}, \tag{S27}$$

where α_{AMPA} and β_{AMPA} are forward and backward binding rates are of AMPAR, and Glu is the maximal concentration of glutamate during the synapse activation.

Time constant τ_{AMPA} of AMPAR activation is defined:

$$\tau_{AMPA} = \frac{1}{\alpha_{AMPA}\, Glu + \beta_{AMPA}}, \tag{S28}$$

The parameters of AMPAR and NMDAR are given in Table S2.

1.4 Partial blockade of Glu2NB-NMDAR restores impaired signal integration in AICD conditions

We performed computational modeling of excitability of CA1 pyramidal neuron in AICD conditions following (Pousinha et al., 2017) (**Fig. S1**). In our study, the stimulation consisted of 10 presynaptic pulses at 10 Hz and 50 Hz. We showed that signal integration and somatic spiking activity were perturbed in AICD conditions, when Glu2NB-NMDAR functioning was enhanced and ion channels of the CA1 pyramidal neuron were modified. Specifically, the conductances of Ca^{2+} - dependent K^+ channels $CagK$ and L-type Ca^{2+} channels CaL, and axonal, somatic and dendritic M-type K^+ channels were increased, and the conductances of axonal, somatic and basal Na^+ channels were descreased.

Partial blockade of Glu2NB-NMDAR restored EPSP amplitude in AICD conditions for 10 Hz stimulation and somatic spike generation at 50 Hz. Partial blockade of Glu2NB-NMDAR prevented overactivation of Ca^{2+}-dependent K^+ channels $CagK$ and allowed normalisation of signal integration in CA1 pyramidal neuron. The results obtained qualitatively reproduced the experimental findings (Pousinha et al., 2017).

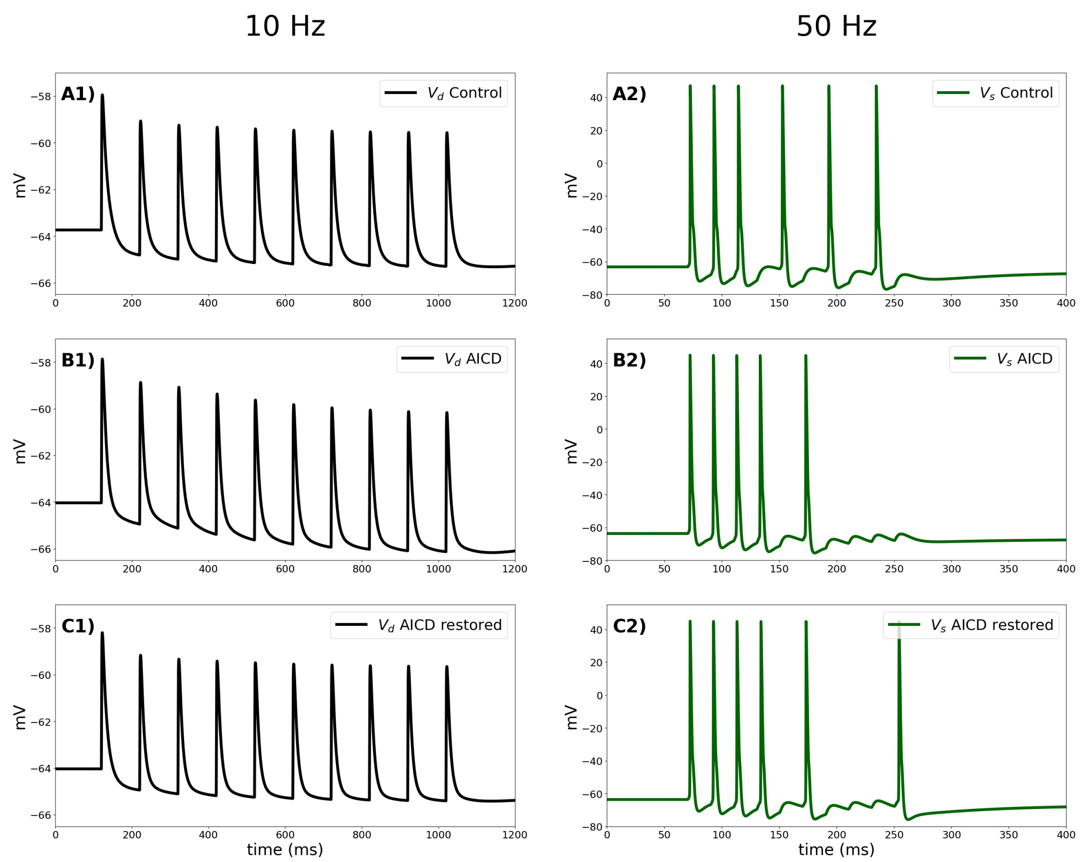

Figure S1. Partial blockade of Glu2NB-NMDAR restores impaired signal integration and spike generation in AICD conditions. Presynaptic inputs were stimulated at 10 Hz (left column) and 50 Hz (right column). GluN2B-NMDAR maximal synaptic conductance was increased by a factor of 4 ($K_{GluN2B} = 4$, $\hat{g}_{NMDA}$=1.6875 x 10^{-2} nS) in AICD conditions. **(A1)** Dendritic membrane potential at a randomly selected synapse in control conditions. AMPAR synaptic conductance $\hat{g}_{AMPA} = 2.4$ x 10^{-1} nS. **(B1)** Dendritic membrane potential at the same randomly selected synapse in AICD conditions. **(C1)** Dendritic membrane potential at the same randomly selected synapse in AICD conditions. Glu2NB-NMDAR is partally blocked leaving 0.25 fraction of its active elevated value. **(A2)** Somatic membrane potential in control conditions. AMPAR synaptic conductance $\hat{g}_{AMPA} = 9$ x 10^{-1} nS. **(B2)** Somatic membrane potential in AICD conditions. **(C2)** Somatic membrane potential in AICD conditions. Glu2NB-NMDAR is partally blocked leaving 0.25 fraction of its active elevated value.

2 SUPPLEMENTARY TABLES

Table S1. Parameters of synaptic plasticity model.

Parameter	Value	Unit	Description
A_{LTP}	8 x 10^{-4}	$1/(mVms)$	Amplitude constant of LTP
A_{LTD}	3.2 x 10^{-2}	$1/(mVms)$	Amplitude constant of LTD
$K_{a1_{LTP}}$	1.7 x 10^{-2}	μS	Value of the filtered $\overline{g}_{NMDA_{LTP}}(t)$ producing half occupation of $\phi_{NMDA_{LTP}}(t)$ for LTP component
$K_{a2_{LTP}}$	1<br>(6.5 x 10^{-2})	μS	Value of the filtered $\overline{g}_{NMDA_{LTP}}(t)$ producing half occupation of $\phi_{NMDA_{LTP}}(t)$ for LTP component inhibition (in parentheses for AICD conditions)
$K_{a_{LTD}}$	3.3 x 10^{-2}	μS	Value of the filtered $\overline{g}_{NMDA_{LTD}}$ producing half occupation of $\phi_{NMDA_{LTD}}(t)$ for LTD component
$\tau_{NMDA_{LTP}}$	100	ms	Time constant of the filtered $\overline{g}_{NMDA_{LTP}}(t)$ for LTP component
$\tau_{NMDA_{LTD}}$	3	ms	Time constant of the filtered $\overline{g}_{NMDA_{LTD}}(t)$ for LTD component
n_{LTP}	4	1	Hill coefficient of $\phi_{NMDA_{LTP}}(t)$ for LTP component
n_{LTD}	2	1	Hill coefficient of $\phi_{NMDA_{LTD}}(t)$ for LTD component
$\tau_{\theta_{LTP}}$	100	ms	Time constant of the moving threshold $\theta_{LTP}(t)$ for LTP component
$\tau_{\theta_{LTD}}$	100	ms	Time constant of the moving threshold $\theta_{LTD}(t)$ for LTD component
$\theta_{V_{LTP}}$	-63	mV	Threshold of $V(t)$ for LTP component
$\theta_{V_{LTD}}$	-63	mV	Threshold of $V(t)$ for LTD component
τ_{LTP}	10	ms	Time constant of the filtered $\overline{V}_{LTP}(t)$ for LTP component
τ_{LTD}	10	ms	Time constant of the filtered $\overline{V}_{LTD}(t)$ for LTD component
τ_{δ}	15	ms	Dirac delta trace time constant
w_{min}	0.2	1	Minimum weight value
w_{max}	2.5	1	Maximum weight value

Table S2. Parameters of NMDAR and AMPAR synapses.

Parameter	Value	Unit	Description	Ref
Glu	1 (1.2)	mM	Glutamate concentration during synapse activation (in parantheses for Aβ conditions)	(Destexhe et al., 1994) (Abramov et al., 2009)
		AMPAR		
α_{GluN2A}	0.5	$/mM/ms$	Forward binding rate of AMPAR	(Destexhe et al., 1994)
β_{AMPA}	0.19	$/ms$	Backward binding rate of AMPAR	(Destexhe et al., 1994)
$\hat{g}_{AMPA}$	2.4 x 10^{-1}	nS	Maximal AMPAR conductance	adjusted
E_{AMPA}	0	mV	AMPA reversal potential	(Destexhe et al., 1994)
		GluN2A-NMDAR and GluN2B-NMDAR		
α_{GluN2A}	0.5	$/mM/ms$	Forward binding rate of GluN2A-NMDAR	fitted (Cull-Candy et al., 2001)
β_{GluN2A}	0.024	$/ms$	Backward binding rate of GluN2A-NMDAR	fitted (Cull-Candy et al., 2001)
α_{GluN2B}	0.1	$/mM/ms$	Forward binding rate of GluN2B-NMDAR	fitted (Cull-Candy et al., 2001)
β_{GluN2B}	0.0075	$/ms$	Backward binding rate of GluN2B-NMDAR	fitted (Cull-Candy et al., 2001)
K_{GluN2A}	1	1	GluN2A-NMDAR scaling factor	adjusted
K_{GluN2B}	1	1	GluN2B-NMDAR scaling factor	adjusted
$\hat{g}_{NMDA}$	9 x 10^{-3}	nS	Maximal NMDAR conductance	adjusted
$[Mg^{2+}]$	1	mM	Extracellular magnesium concentration	(Destexhe et al., 1994)
E_{NMDA}	0	mV	NMDA reversal potential	(Destexhe et al., 1994)

REFERENCES

Abramov, E., Dolev, I., Fogel, H., Ciccotosto, G. D., Ruff, E., and Slutsky, I. (2009). engAmyloid-beta as a positive endogenous regulator of release probability at hippocampal synapses. *Nature Neuroscience* 12, 1567–1576. doi:10.1038/nn.2433

Andrade-Talavera, Y., Duque-Feria, P., Paulsen, O., and Rodríguez-Moreno, A. (2016). Presynaptic Spike Timing-Dependent Long-Term Depression in the Mouse Hippocampus. *Cerebral Cortex (New York, N.Y.: 1991)* 26, 3637–3654. doi:10.1093/cercor/bhw172

Cull-Candy, S., Brickley, S., and Farrant, M. (2001). NMDA receptor subunits: Diversity, development and disease. *Current Opinion in Neurobiology* 11, 327–335. doi:10.1016/s0959-4388(00)00215-4

Dainauskas, J. J., Marie, H., Migliore, M., and Saudargiene, A. (2023). GluN2B-NMDAR subunit contribution on synaptic plasticity: A phenomenological model for CA3-CA1 synapses. *Frontiers in Synaptic Neuroscience* 15, 1113957. doi:10.3389/fnsyn.2023.1113957

Destexhe, A., Mainen, Z. F., and Sejnowski, T. J. (1994). Synthesis of models for excitable membranes, synaptic transmission and neuromodulation using a common kinetic formalism. *Journal of Computational Neuroscience* 1, 195–230. doi:10.1007/BF00961734

Migliore, R., Lupascu, C. A., Bologna, L. L., Romani, A., Courcol, J.-D., Antonel, S., et al. (2018). The physiological variability of channel density in hippocampal CA1 pyramidal cells and interneurons explored using a unified data-driven modeling workflow. *PLOS Computational Biology* 14, e1006423. doi:10.1371/journal.pcbi.1006423

Morishita, W., Lu, W., Smith, G. B., Nicoll, R. A., Bear, M. F., and Malenka, R. C. (2007). Activation of NR2B-containing NMDA receptors is not required for NMDA receptor-dependent long-term depression. *Neuropharmacology* 52, 71–76. doi:10.1016/j.neuropharm.2006.07.005

Peng, Y., Lu, Z., Li, G., Piechowicz, M., Anderson, M., Uddin, Y., et al. (2016). The autism-associated MET receptor tyrosine kinase engages early neuronal growth mechanism and controls glutamatergic circuits development in the forebrain. *Molecular Psychiatry* 21, 925–935. doi:10.1038/mp.2015.182

Pousinha, P. A., Mouska, X., Bianchi, D., Temido-Ferreira, M., Rajão-Saraiva, J., Gomes, R., et al. (2019). The Amyloid Precursor Protein C-Terminal Domain Alters CA1 Neuron Firing, Modifying Hippocampus Oscillations and Impairing Spatial Memory Encoding. *Cell Reports* 29, 317–331.e5. doi:10.1016/j.celrep.2019.08.103

Pousinha, P. A., Mouska, X., Raymond, E. F., Gwizdek, C., Dhib, G., Poupon, G., et al. (2017). Physiological and pathophysiological control of synaptic GluN2B-NMDA receptors by the C-terminal domain of amyloid precursor protein. *eLife* 6. doi:10.7554/eLife.25659
